# Chronic Exposure to Both Electronic and Conventional Cigarettes Alters Ileum and Colon Turnover, Immune Function, and Barrier Integrity in Mice

**DOI:** 10.3390/jox14030053

**Published:** 2024-07-22

**Authors:** Madjid Djouina, Anaïs Ollivier, Christophe Waxin, Gwenola Kervoaze, Muriel Pichavant, Ségolène Caboche, Djamal Achour, Céline Grare, Delphine Beury, David Hot, Sébastien Anthérieu, Jean-Marc Lo-Guidice, Laurent Dubuquoy, David Launay, Cécile Vignal, Philippe Gosset, Mathilde Body-Malapel

**Affiliations:** 1 Univ. Lille, Inserm, CHU Lille, U1286-INFINITE—Institute for Translational Research in Inflammation, F-59000 Lille, France; madjid.djouina@univ-lille.fr (M.D.); christophe.waxin@univ-lille.fr (C.W.); laurent.dubuquoy@inserm.fr (L.D.); david.launay@univ-lille.fr (D.L.); cecile.vignal2@univ-lille.fr (C.V.); 2 Univ. Lille, CNRS, INSERM, Institut Pasteur de Lille, CHU Lille, Center for Infection and Immunity of Lille (CIIL), UMR9017-U1019, F-59000 Lille, France; anais.ollivier@univ-lille.fr (A.O.); gwenola.kervoaze@univ-lille.fr (G.K.); muriel.pichavant@pasteur.fr (M.P.); philippe.gosset@pasteur-lille.fr (P.G.); 3 Univ. Lille, CNRS, Inserm, CHU Lille, Institut Pasteur de Lille, US41-UAR 2014-PLBS, F-59000 Lille, France; segolene.caboche@univ-lille.fr (S.C.); delphine.beury@univ-lille.fr (D.B.); david.hot@pasteur-lille.fr (D.H.); 4 Univ. Lille, CHU Lille, Institut Pasteur de Lille, ULR 4483-IMPECS—IMPact de l’Environnement Chimique sur la Santé, F-59000 Lille, France; djamal.achour@univ-lille.fr (D.A.); celine.grare@univ-lille.fr (C.G.); sebastien.antherieu@univ-lille.fr (S.A.); jean-marc.lo-guidice@univ-lille.fr (J.-M.L.-G.)

**Keywords:** e-cigarette, intestinal, immune response, proliferation, permeability, microbiota

## Abstract

Although the effects of cigarette smoke (CS) on the development of several intestinal diseases is well documented, the impact of e-cigarette aerosol (e-cig) on digestive health is largely unknown. To compare the effects of e-cig and CS on mouse ileum and colon, animals were chronically exposed for 6 months by nose-only inhalation to e-cig at 18 or 30 W power, or to 3R4F CS. Results showed that e-cig exposure decreased colon cell proliferation. Several other proliferative defects were observed in response to both e-cig and CS exposure, including up- and down-regulation of cyclin D1 protein levels in the ileum and colon, respectively. E-cig and CS exposure reduced myeloperoxidase activity in the ileum. In the colon, both exposures disrupted gene expression of cytokines and T cell transcription factors. For tight junction genes, ZO-1- and occludin-protein expression levels were reduced in the ileum and colon, respectively, by e-cig and CS exposure. The 16S sequencing of microbiota showed specific mild dysbiosis, according to the type of exposure. Overall, e-cig exposure led to altered proliferation, inflammation, and barrier function in both the ileum and colon, and therefore may be a gut hazard on par with conventional CS.

## 1. Introduction

Backed by strong epidemiological evidence, research shows that cigarette smoke (CS) disturbs gut homeostasis and influences the development of several intestinal pathologies such as colorectal cancer (CRC), microscopic colitis, Crohn’s disease (CD), and ulcerative colitis (UC). For example, a recent meta-analysis demonstrated that CS increases the risk of CRC in a dose-dependent manner with intensity and duration [1]. A systematic review and meta-analysis by Jaruvongvanich et al. found a significantly higher risk of microscopic colitis among current smokers compared with never-smokers [2]. Furthermore, the risk was attenuated among former smokers but remained significantly higher among never-smokers. CS has a dichotomous effect in inflammatory bowel diseases (IBDs). A 2019 umbrella review of meta-analyses identified smoking as the strongest risk factor for CD [3]. Current smokers had an increased risk of CD (OR, 1.76; 95% CI, 1.40–2.22) [3]. Another meta-analysis confirmed that CS increases the risk of surgery in patients with IBD [4]. Current smoking and former smoking are important risk factors for intestinal resection among patients with CD and colectomy among patients with UC, respectively [4]. Paradoxically, CS is a protective factor for the development and progression of UC [5]. Current smokers had decreased risk of UC (OR, 0.58; 95% CI, 0.45–0.75) compared with never-smokers [3].

Several underlying mechanisms have been proposed to explain the effects of CS on intestinal diseases. Nicotine induces systemic innate and adaptive immunosuppression [6]. CS impairs the mucosal immune response, including causing an increased production of cytokines and proteases by Th17 cells, innate lymphoid cells 3, neutrophils [7], invariant natural killer T (iNKT) cells [8], and activated macrophages [9]. The arachidonic acid-metabolising enzymes cytochrome c oxidase subunit 2 and polyunsaturated fatty acid 5-lipoxygenase mediate tumour development in mouse CS-induced CRC [10,11,12]. CS exposure caused systemic and intestinal ischaemia which led to angiogenesis and gastrointestinal epithelial barrier dysfunction, which could contribute to the increased risk and severity of CD in smokers [13].

Several lines of evidence have shown that CS exposure affects gut barrier function in mice. CS damaged the morphology of intestinal epithelial cells and impaired the expression of proteins involved in tight junction proteins zonula occludens-1 (ZO-1) and occludin [14,15]. This effect of CS exposure is also related to alteration in the expression of mucin, the main component of the mucus layer, which plays a critical role in gut barrier function. In the ileum, relative mRNA expression of *Muc2* and *Muc3* were significantly increased, whereas in the colon an increased expression of *Muc4* was observed [16]. Additionally, a functional permeability defect and an increase in bacterial translocation in mesenteric and caudal lymph nodes have been shown following CS exposure [14,15].

CS seems to alter gut microbiota composition, inducing dysbiosis. Disturbance of gut microbiota diversity and composition have been repeatedly described in mice and humans [17,18,19]. However, the features of dysbiosis are not always consistent among studies. These discrepancies can be explained by a lack of strategies to take into account confounding factors and to accurately quantify CS exposure (e.g., indoor vs. outdoor, active vs. passive smoking, number of cigarettes/day, etc.), which have weakened the strength of the findings [18,20]. In mice, it has been shown that smoke-induced gut microbiota dysbiosis plays a protumourigenic role in CRC through alteration of gut metabolites and impairment of gut barrier function, which could activate oncogenic MAPK/ERK signalling in the colonic epithelium [21]. Overall, the effects of CS toxicants on intestinal microbiota are not yet clearly defined.

In addition to conventional cigarette smoking, the use of e-cigarettes or ‘vaping’ has become common, particularly among teenagers and smokers who are trying to quit. E-cigarettes are presented as a less-damaging nicotine delivery system or as a new smoking-cessation tool. Many e-cigarette users believe that CS is less healthy than vaping. But even though the toxic effects of conventional cigarette products have been extensively researched, the long-term health effects of e-cigarette aerosol (e-cig) have not been well studied, particularly at the intestinal level. A retrospective case-control study of patients with CD or UC failed to find an association between current e-cigarette use and worse outcomes among patients with IBD [22]. Nevertheless, an in vivo and ex vivo study reported that e-cig led to a compromised gut barrier and was associated with chronic gut inflammation [23]. In rats, e-cig exposure for 4 weeks induced oxidative stress, colonic mucosa epithelial loss, and inflammatory infiltration, as well as a decrease in goblet cells [24]. In mice, daily inhalation of mango-flavoured e-cig for one month was associated with colon inflammation [25]. Further investigation of the impact of CS and e-cig is therefore necessary.

In this study, we aimed to assess the in vivo intestinal effects of e-cig chronic exposure in comparison to CS. For this, mice were subjected to e-cig and CS exposure for 6 months. Ileum and colon inflammation, cell proliferation, and permeability parameters were assessed and colon luminal microbiota were analysed.

## 2. Materials and Methods

### 2.1. E-Cigarettes and Conventional Cigarettes

Today there is a wide variety of e-cigarettes and e-liquids available for purchase. As described in detail previously [26,27], the third generation “Mod Box” model (SCIREQ^®^, Emka Technologies, Paris, France) was chosen, which uses an “Air Tank” clearomiser equipped with a 0.5 Ω Kanthal coil and with a partially closed air flow. Two power settings for the Mod Box model were chosen: a “low” power of 18 W and a “high” power of 30 W, thereafter called Mb18W and Mb30W. Both devices are from NHOSS^®^ (Innova, Bondues, France). For the e-liquid, we chose the best-selling NHOSS brand containing 65% propylene glycol, 35% glycerine and 16 mg/mL nicotine, and the most common flavour, “blond tobacco”, representative of a standard e-liquid in accordance with the French national organisation for standardisation (AFNOR, Association Française de Normalisation) recommendations from 2015. Conventional 3R4F cigarettes were obtained from the University of Kentucky (Lexington, KY, USA).

### 2.2. Aerosol Generation and Mice-Exposure Protocols

The experimental protocol was previously described [28]. To avoid chemical cross-contamination, two different pieces of equipment (exposure towers and pipes) were used for e-cig and 3R4F exposures. Aerosols from e-cig were generated with an InExpose e-cigarette extension system on which we adapted the Mod Box. Another InExpose system with a cigarette-smoking robot (SCIREQ, Emka Technologies, Paris, France) was used to generate 3R4F CS. Mice were exposed to e-cig or CS by using separate nose-only towers (InExpose system, SCIREQ, Emka Technologies). In order to perform a comparative study of the intestinal effects of the e-cig and CS, a 6-month chronic exposure was performed (60 min/day, 5 days/week for e-cig and 3R4F), with all products tested with Health Canada Intense puff profile (55 mL puff volume, 2 s puff duration, 30 s puff period). One group was sham-exposed to fresh conditioned air (the negative control; thereafter called the Air group). Therefore, mice were exposed for 6 months, 5 days per week, to 120 puffs/day. These exposure levels are similar to those used in other murine studies (150 and 120 puffs daily [29,30]). Moreover, this level of e-cig exposure can be estimated as relevant to human exposure, examples of which recently reached 373 ± 125 puffs per day in e-cig modifiable device users from Maryland [31]. Our chemical characterization of these models showed emission of carbonyls compounds (formaldehyde, acetaldehyde, crotonaldehyde, and methyl ethyl ketone) and polycyclic aromatic hydrocarbons (naphthalene, phenanthrene, fluoranthene, and pyrene) by e-cig and CS, higher for CS than e-cig [27]. Moreover, the Modbox model was shown to deliver 60 μg nicotine/puff at 18 W setting and 137 μg nicotine/puff at 30 W setting. In comparison, the 3R4F cigarette delivered 95 µg nicotine/puff, under the HCI puffing regime [27].

Two independent experiments were conducted on 9-week-old, male BALB/c mice (Janvier Labs, Le Genest Saint Isle, France) using 7 animals per group. A first set of experiments was performed with 7 mice in the Air group, 7 mice in the Mb18W e-cig group, 7 mice in the Mb30W e-cig group, and 7 mice in the 3R4F CS group. An identical experiment was then carried out. Thus, a total of 14 mice per group were analysed.

### 2.3. Serum Cotinine Levels

Following necropsy, mice sera were obtained from blood samples by centrifugation at 3500× *g* for 20 min and stored at −80 °C for subsequent assays. The plasma cotinine level was assayed using the Cotinine ELISA Kit (# KA0930, Abnova, Heidelberg, Germany).

### 2.4. Quantitative RT-PCR

Quantitative RT-PCR was performed in small-intestinal and colonic tissue samples according to a previously described method detailed in the Supplementary Method File [32].

### 2.5. Histological Analysis

Epithelial area in the colon, villus height, and crypt depth in the proximal and distal small intestine were measured according to a previously described method detailed in the Supplementary Method File [33].

### 2.6. Immunohistochemical Staining and Quantification

Serial histological sections of 4 µm thickness were cut, deparaffinised, and rehydrated. For apoptosis analysis in ileum and colon sections based on the terminal deoxynucleotidyl transferase-mediated dUTP nick end labelling (TUNEL) assay, the In Situ Cell Death Detection Kit, TMR red TUNEL kit (#12156792910, Roche, Boulogne-Billancourt, France) was used according to the manufacturer’s specifications. Tissue sections were pretreated in permeabilisation solution (0.1% Triton X-100, 0.1% sodium citrate) for 8 min. After washing, the samples were labelled with the TUNEL reaction mixture and incubated for 60 min at 37 °C in a humidified atmosphere in the dark. Sections were counterstained with DAPI (Molecular Probes, Fisher Scientific, Illkirch, France).

For immunohistochemistry, antigen retrieval was performed using the heat-induced antigen retrieval method for Ki-67, cyclin D1, and occludin with 10 mM sodium citrate buffer in a decloaking chamber for 20 min at 121 °C. For ZO-1, the Proteinase K Antigen Retrieval method was used where sections were incubated with a solution containing 10 µg/mL of proteinase K in TE buffer (50 mM Tris Base, 1 mM EDTA, 0.5% Triton X-100; pH 8.0) for 10 min at 37 °C. After washing, all sections were blocked for 30 min with 5% goat serum. Rabbit anti-mouse Ki-67 primary antibody (MA5-14520, Invitrogen; Fisher Scientific, Illkirch, France 1/100 dilution), rabbit anti-mouse cyclin D1 primary antibody (#55506, Cell Signaling Technology; Danvers, USA 1/400 dilution), rabbit anti-mouse ZO-1 primary antibody (#61-7300, Invitrogen; 1/200 dilution), and rabbit anti-mouse occludin primary antibody (#91131, Cell Signaling; 1/200 dilution) were incubated overnight at 4 °C.

For cyclin D1 and ZO-1 staining, tissue sections were incubated for 30 min at room temperature with the ImmPRESS^®^ HRP Goat Anti-Rabbit IgG Polymer Detection Kit (MP-7451, Vector Laboratories, Peterborough, UK), and staining was developed using SignalStain^®^ DAB Substrate (#8059, Cell Signaling Technology, Danvers, MA, USA). Nuclear staining with haematoxylin was performed before adding the mounting medium.

For Ki-67 and occludin staining, tissue sections were incubated with anti-rabbit Alexa Fluor 488 conjugated secondary antibody (A-21206, Invitrogen; 1/400 dilution) for 1 h. Sections were counterstained with DAPI.

Slides were scanned using a Zeiss AxioScan.Z1^®^ slide scanner (Zeiss, Fougères, France) at 20X magnification, allowing the analysis of the whole tissue sections. Image analysis was conducted using QuPath software (version 0.4.3) [34]. The percentage of apoptotic cells (TUNEL-positive staining) and proliferating cells (Ki-67-positive staining) was determined using the “Positive Cell Detection” command. Additionally, optical density for cyclin D1 and ZO-1, as well as fluorescence intensity for occludin, were measured using the “Cell Intensity Measurements” function.

### 2.7. Myeloperoxidase (MPO) Activity

MPO is a protein found in granules of neutrophils and its quantification is used as a marker of inflammation. Smal- intestine and colon specimens were homogenised with a Precellys^®^ 24 homogeniser in a phosphate buffer (pH 6.0) containing 0.5% hexadecyltrimethylammonium bromide (Sigma-Aldrich, Merck, Darmstadt, Germany) and subjected to two sonication and freeze-thaw cycles. The suspensions were centrifuged at 14,000× *g* for 15 min at 4 °C and the supernatants were reacted with 1 mg/mL o-dianisidine hydrochloride and 0.0005% hydrogen peroxide. Optical density of each sample was read at 450 nm with a Versamax microplate reader (Molecular Devices, San Jose, USA). One unit of MPO activity was defined as the amount that degraded 1 µmol peroxidase per min at 25 °C. The results were expressed as absorbance per total quantity of proteins determined using the DCTM protein assay kit (Bio-Rad, Marnes-la-Coquette, France).

### 2.8. Bacterial DNA Extraction and Illumina MiSeq Sequencing

They were performed from genomic DNA extracted from colon luminal content, according to a previously described method detailed in the Supplementary Method File [32].

### 2.9. Analysis of Sequencing Data

Bioinformatic analyses were performed according to a previously described method detailed in the Supplementary Method File [32].

### 2.10. Statistics

Results are expressed as mean ± standard error of the mean. The statistical significance of differences between experimental groups was calculated using the Mann–Whitney nonparametric U test (GraphPad Prism software v8). Statistical significance was defined as *p* < 0.05 for all experiments, with * *p* < 0.05, ** *p* < 0.01, *** *p* < 0.005, and **** *p* < 0.001 vs. the Air group.

## 3. Results

### 3.1. Effects of e-Cig and CS Exposure on Ileum and Colon Histomorphology, Apoptosis, and Cell Proliferation

**Figure 1:**
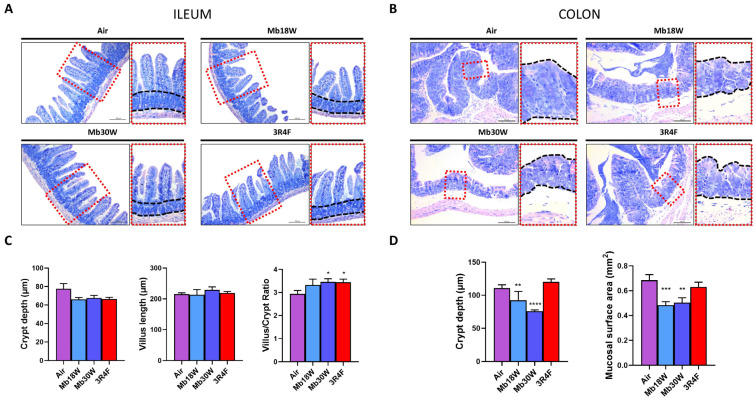
Effects of chronic electronic and conventional cigarette exposure on ileum and colon histomorphology. (**A**) Representative images of May Grünwald Giemsa (MGG)-stained ileum sections, with higher magnification on the right. (**B**) Representative images of MGG-stained colon sections, with higher magnification on the right. (**C**) Measurement of ileum crypt depth, villus length, and villus/crypt ratio. (**D**) Measurement of colon crypt depth and mucosal surface area. n = 10 per group. * *p* < 0.05, ** *p* < 0.01, *** *p* < 0.005 and **** *p* < 0.001 compared to the control group (Air), as determined by the Mann–Whitney U test.

Male mice were exposed for 6 months to Mod Box e-cig at two power settings (Mb18W and Mb30W groups), CS (3R4F group), or conditioned air (Air group) by nose-only inhalation. First, levels of cotinine, a major metabolite and surrogate marker for nicotine, were measured [35]. As shown in Supplementary Figure S1, plasma cotinine levels gradually and significantly increased in Mb18W-, Mb30W-, and 3R4F-exposed mice compared to Air-exposed mice (*p* < 0.0001). Gut mucosal structure was assessed in MGG-stained sections of ileum and colon (Figure 1A,B). In the ileum, villus length and crypt depth did not vary between the groups, although there was a trend to a lower crypt depth in Mb18W-, Mb30W-, and 3R4F-exposed mice (Figure 1C). The villus length/crypt depth ratio was significantly increased in Mb30W- and 3R4F-exposed mice, suggesting a dysregulation of ileal cell proliferation. In the colon, the crypt depth and the mucosal surface area were measured (Figure 1D). In contrast with CS-exposed mice, both parameters were reduced in Mb18W- and Mb30W-exposed mice, suggesting a decrease in colon cell proliferation by e-cig exposure.

**Figure 2:**
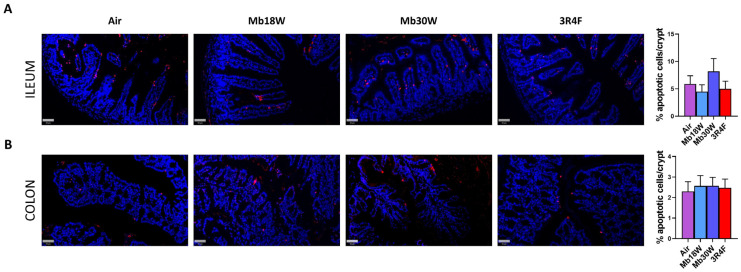
Effects of chronic electronic and conventional cigarette exposure on ileum and colon apoptosis. Terminal deoxynucleotidyl transferase-mediated dUTP nick end labelling (TUNEL) immunostaining and quantification of apoptotic cells per crypt in ileum (**A**) and colon (**B**). TUNEL stain: red. Nuclear stain: blue. n = 6 per group. Scale bar = 50 µm.

The TUNEL assay was used to determine whether the histomorphological impairments may be linked with apoptosis. The TUNEL assay detects DNA breakage by labelling free 3′-hydroxyl termini. Because genomic DNA breaks occur during early and late stages of apoptosis, TUNEL staining continues to be widely used to measure apoptotic cell death. In our study, the number of apoptotic cells per crypt was not modified in either the ileum or the colon by e-cig and CS exposure (Figure 2A,B).

**Figure 3:**
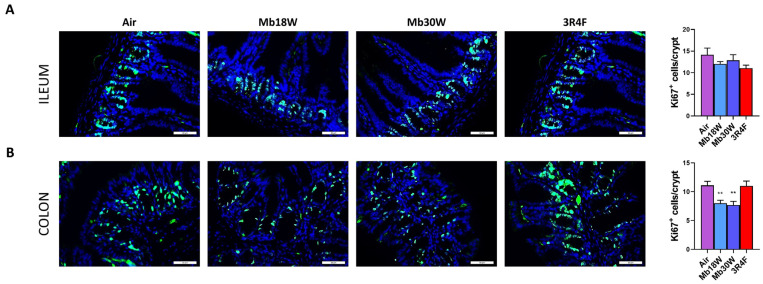
Effects of chronic electronic and conventional cigarette exposure on ileum and colon proliferation. Ki-67 immunostaining and quantification of Ki-67-positive cells per crypt in ileum (**A**) and colon (**B**). Ki67 stain: green. Nuclear stain: blue. n = 6 per group. ** *p* < 0.01 compared to the control group (Air), as determined by the Mann–Whitney U test. Scale bar = 50 µm.

The effects of e-cig and CS inhalation were evaluated on ileum- and colon-cell proliferation. Whereas there was no change in the ileum (Figure 3A), exposure to Mb18W and Mb30W reduced the number of Ki-67-positive cells per crypt in the colon, suggesting that the crypt loss was caused by decreased cell proliferation in the colon (Figure 3B). CS did not modify the Ki-67-positive cell number in either the ileum or colon.

**Figure 4:**
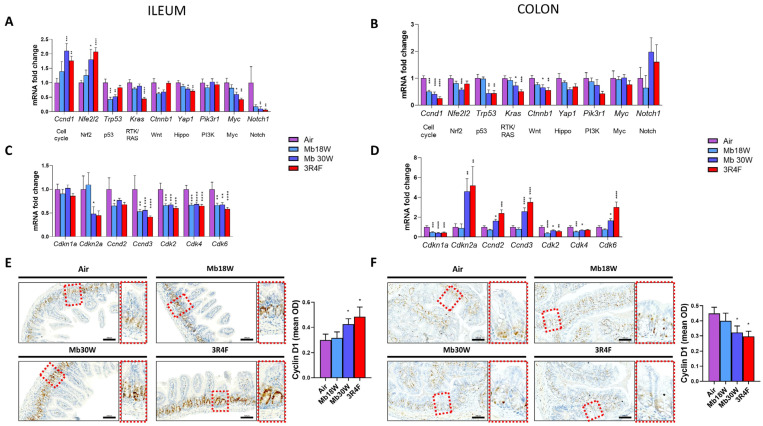
Effects of chronic electronic and conventional cigarette exposure on ileum- and colon-proliferation signalling pathways. Transcript levels of markers of major proliferation pathways in ileum (**A**) and colon (**B**). n = 14 per group. Transcript levels of cyclin D1 pathway genes in ileum (**C**) and colon (**D**). n = 14 per group. Cyclin D1 immunostaining, with higher magnification on the right, and quantification of cyclin D1 mean optical density in ileum (**E**) and colon (**F**). Cyclin D1 stain: brown. Nuclear stain: blue. n = 5 per group. * *p* < 0.05, ** *p* < 0.01, *** *p* < 0.005 and **** *p* < 0.001 compared to the control group (Air), as determined by the Mann–Whitney U test.

To further explore the proliferation defects observed above, prototypical markers of the main proliferation pathways were selected based on Sanchez-Vega et al. [36] and quantified by RT-qPCR. In the ileum, Mb18W exposure downregulated *Trp53* and *Ctnnb1* mRNA levels. Mb30W aerosol inhalation induced upregulation of *Ccnd1* and *Nfe2l2*, and downregulation of *Trp53*, *Yap1*, *Myc*, and *Notch1* (Figure 4A). 3R4F smoke inhalation upregulated *Ccnd1* and *Nfe2l2* and downregulated *Kras*, *Yap1*, *Myc*, and *Notch1* ileum transcript levels. In the colon, *Ccnd1* mRNA was downregulated by MB18W exposure, and levels of *Nfe2l2*, *Trp53*, *Kras*, and *Ctnnb1* were lower following Mb30W exposure (Figure 4B). A similar pattern was observed after CS inhalation: *Ccnd1*, *Trp53*, *Kras*, and *Ctnnb1* also showed significantly reduced levels.

Since *Ccnd1* transcript variation was detected in both the ileum and colon, and following both e-cig and CS exposure, we further explored the cell cycle pathway (Figure 4C,D). The relative expression of several components of the cell cycle pathway were found modulated in the ileum and colon of intoxicated groups. Ileal *Cdkn2a* and *Ccnd2* transcripts were reduced by Mb30W and Mb18W exposures, respectively (Figure 4C). *Ccnd3*, *Cdk2*, *Cdk4*, and *Cdk6* mRNA levels were all greatly downregulated in the ileum of Mb18W-, Mb30W-, and 3R4F-exposed mice. The mRNA relative expression of *Cdkn2a*, *Ccnd2*, *Ccnd3*, and *Cdk6* were increased in the colon of Mb30W- and 3R4F-exposed mice (Figure 4D). *Cdk4* transcripts were reduced by Mb18W and Mb30W inhalation, and *Cdkn1* and *Cdk2* were downregulated by both 18W and 30W e-cig exposure, as well as CS.

Lastly, cyclin D1 immunohistochemical staining was performed (Figure 4E,F). As observed for the gene transcript levels, measurement of cyclin D1 staining intensity demonstrated that Mb30W and 3R4F exposure significantly increased cyclin D1 protein levels in the ileum, and, in contrast decreased, them in the colon (Figure 4E,F). Overall, our results showed that proliferation signalling is impaired in the ileum and colon by e-cig and CS exposure, particularly in the cell cycle pathway, and these disturbances are associated with increased and decreased cyclin D1 protein expression the in ileum and colon, respectively.

### 3.2. Inflammatory Response to e-Cig and CS Exposure

**Figure 5:**
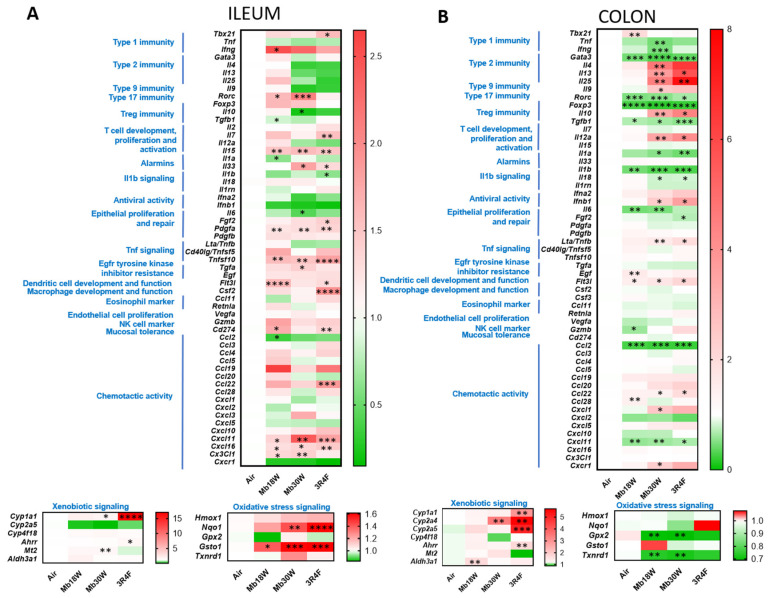
Effects of chronic electronic and conventional cigarette exposure on ileum and colon inflammatory patterns. (**A**) Transcript levels of major inflammatory cytokines, chemokines, T cell transcription factors, xenobiotic metabolism and oxidative stress markers in ileum. (**B**) The same respective parameters in the colon. n = 14 per group. * *p* < 0.05, ** *p* < 0.01, *** *p* < 0.005, and **** *p* < 0.001, compared to the control group (Air), as determined by the Mann–Whitney U test.

Gene transcript levels of inflammatory cytokines and chemokines, transcription factors representative of the Th1, Th2, Th17, and Treg immune responses, xenobiotic metabolism and oxidative stress markers were quantified in the ileum and colon to assess whether e-cig and CS exposure impairs the intestinal inflammatory response (Figure 5).

In the ileum, Mb18W exposure induced an upregulation of interferon gamma *Ifng*, FMS-like tyrosine kinase 3 ligand *Flt3l* and CD274 antigen *Cd274* (also called Pdl1) and a downregulation of transforming growth factor beta 1 *Tgfb1*, interleukin 1 alpha *Il1a*, and C-C motif chemokine ligand 2 *Ccl2* (also called MCP-1) expression compared to control mice (Figure 5A). Mb30W exposure decreased *Il10* and *Il60* and increased *Il33*, *Tgfa*, Cytochrome P450 1A1 *Cyp1a1*, Metallothionein 2 *Mt2*, and NAD(P)H dehydrogenase quinone 1 *Nqo1* mRNA levels. Both Mb18W and Mb30W upregulated the Th17 transcription factor nuclear receptor ROR-gamma *Rorc*, *Il15*, platelet-derived growth factor subunit A *Pdgfa*, tumor necrosis factor ligand superfamily member 10 *Tnfsf10* (also called Trail), C-X-C motif chemokine 11 *Cxcl11*, *Cxcl16*, Fractalkine *Cx3cl1* and glutathione S-transferase omega-1 *Gsto1* expression. Exposure to CS decreased *Il1b* and increased the Th1 transcription factor T-box transcription factor TBX21 *Tbx21* (also called T-bet), *Il7*, *Il15*, *Il33*, fibroblast growth factor 2 *Fgf2*, *Pdgfa*, *Tnfsf10*, *Flt3l*, granulocyte-macrophage colony-stimulating factor *Csf2*, *Cd274*, *Ccl22*, *Cxcl11*, *Cxcl16*, *Cyp1a1*, Aryl hydrocarbon receptor repressor *Ahrr*, *Nqo1*, and *Gsto1* transcription. Overall, our data argue in favour of increased ileal chemotactic activity and oxidative stress induced by both e-cig and CS. By contrast, activity of MPO, a well-established neutrophil marker and an important regulator of immune system function [37], was quantified. MPO activity was decreased by both Mb30W e-cig and CS exposure in the ileum (Supplementary Figure S2).

The same parameters representative of inflammatory responses were quantified in the colon. Some markers were modulated only in the Mb18W-exposed mice such as *Tbx21*, pro-epidermal growth factor *Egf*, *Ccl28*, and aldehyde dehydrogenase dimeric NADP-preferring *Aldh3a1*, which were upregulated, and granzyme B *Gzmb*, which was downregulated (Figure 5B). Other markers were modulated only in the Mb30W-exposed mice such as *Tnf*, *Ifng*, *Il1a*, and *Il18*, which were downregulated, and *Il4*, *Il13*, *Il25*, *Il9*, *Il10*, *Il12a*, *Ifnb1*, lymphotoxin A *Lta* (also called *Tnfb*), *Ccl22*, *Cxcl1*, C-X-C chemokine receptor 1 *Cxcr1* (also called *Il8ra*) and *Cyp2a4*, which were upregulated. The trans-acting T-cell-specific transcription factor GATA-3 *Gata3*, *Rorc*, forkhead box protein P3 *Foxp3*, *Tgfb1*, *Il1b*, *Il6*, *Ccl2*, *Cxcl11*, glutathione peroxidase 2 *Gpx2* and thioredoxin reductase 1 *Txnrd1* relative expression was decreased, and *Flt3l* relative expression was increased in both Mb18W- and Mb30W-exposed mice. *Gata3*, *Rorc*, *Foxp3*, *Tgfb1*, *Il1a*, *Il1b*, *Il18*, *Fgf2*, *Ccl2*, and *Cxcl11* levels were reduced in 3R4F-exposed mice. *Il13*, *Il25*, *Il10*, *Il12a*, *Ifnb1*, *Lta*/*Tnfb*, *Flt3l*, *Ccl22*, *Cyp1a1*, *Cyp2a4*, *Cyp2a5*, and *Ahrr* levels were enhanced in 3R4F-exposed mice. Noteworthily, a strong downregulation of the Th1, Th17, and Treg transcription factors, namely *Gata3*, *Rorc*, and *Foxp3*, respectively, were observed following exposure to e-cig and CS. No differences were observed in colon MPO activity in any condition (Supplementary Figure S2). In summary, the immune response presented numerous disturbances in mice exposed to e-cig and CS, higher in the colon than in the ileum.

### 3.3. Effects of e-Cig and CS Exposure on Ileum- and Colon-Permeability Markers

To assess gut barrier function, mRNA expression levels of occludin (*Ocln*), tight junction protein ZO-1 (*Tjp1*), junctional adhesion molecule A (*F11r*), and cadherin 1 (*Cdh1*) were measured.

**Figure 6:**
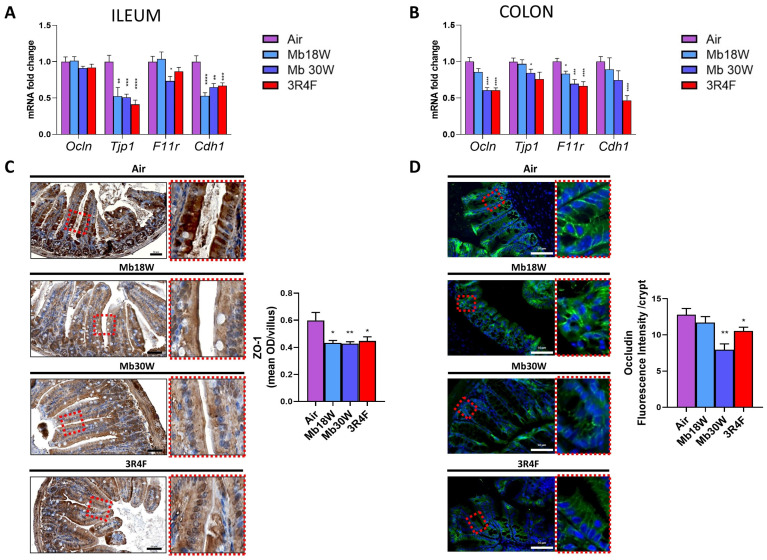
Effects of chronic electronic and conventional cigarette exposure on ileum and colon permeability. Transcript levels of major permeability markers in ileum (**A**) and colon (**B**). n = 14 per group. (**C**) ZO-1 immunostaining, with higher magnification on the right, and quantification of ZO-1 mean optical density in ileum. ZO-1 stain: brown. Nuclear stain: blue. n = 6 per group. (**D**) Occludin immunostaining, with higher magnification on the right, and quantification of occludin mean fluorescent intensity in the colon. Occludin stain: green. Nuclear stain: blue. n = 6 per group. * *p* < 0.05, ** *p* < 0.01, *** *p* < 0.005, and **** *p* < 0.001 compared to the control group (Air,) as determined by the Mann–Whitney U test.

In the ileum, decreased levels of *F11r* were found in Mb30W-exposed mice, and reduced levels of *Tjp1* and *Cdh1* were observed following Mb18W, Mb30W, and 3R4F exposure (Figure 6A). In the colon, 3R4F exposure downregulated the expression of *Ocln*, *F11r*, and *Cdh1* (Figure 6B). Relative levels of *Ocln* and *Tjp1* were reduced by Mb30W exposure, and *F11r* levels were diminished in both Mb18W and Mb30W groups. To explore if these transcriptional disturbances were associated with a decrease in junction protein, we performed immunohistochemical staining of the two most impaired proteins by e-cig and CS in the ileum and colon, respectively, ZO-1 and occludin. In the ileum, we confirmed that *Tjp1* mRNA downregulation was associated with ZO-1 protein under-expression (Figure 6C). Moreover, in the colon, the downregulation of *Ocln* transcripts levels was corroborated by decreased levels of occludin protein following Mb30W and CS exposure (Figure 6D). In conclusion, our data suggest that both vaping and conventional smoking compromised barrier integrity in the ileum and colon.

### 3.4. Effects of e-Cig and CS Exposure on Gut Microbiota

To assess the effect of e-cig and CS exposure on gut microbiota, the diversity and composition of the bacterial community were analysed with 16S rRNA amplicon sequencing of colon luminal content.

**Figure 7:**
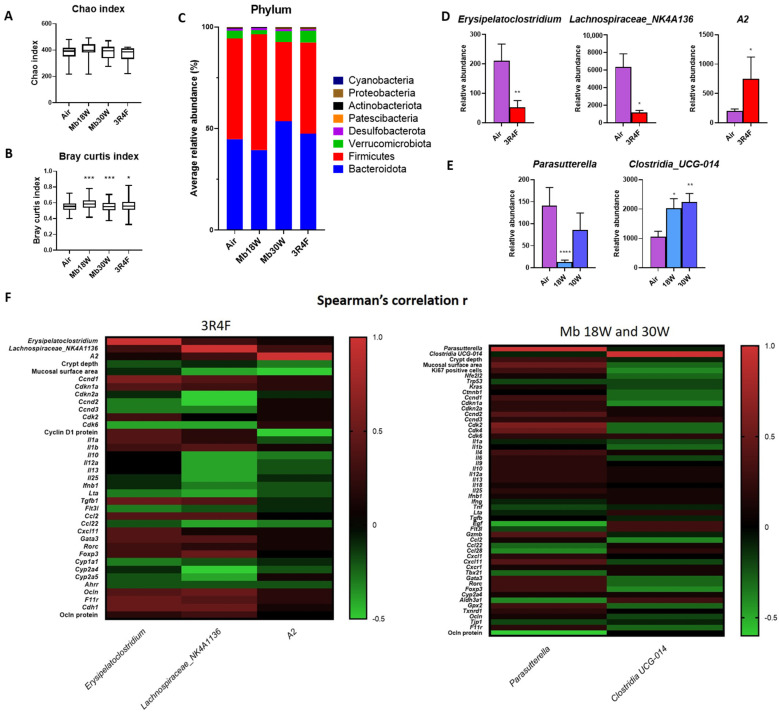
Effects of chronic electronic and conventional cigarette exposure on gut microbiota. n = 14 per group. (**A**) Box plots representing Chao α-diversity index. (**B**) Box plots representing Bray–Curtis β-diversity index. * *p* < 0.05, *** *p* < 0.005, as determined by the Kruskal–Wallis test. (**C**) Differential abundance of bacterial phyla. (**D**) Differential abundance of significantly changed bacterial genera in the 3R4F group compared to the control Air group. (**E**) Differential abundance of significantly changed bacterial genera in e-cigarette-exposed groups compared to the Air group. (**D**,**E**) * *p* < 0.05, ** *p* < 0.01, and **** *p* < 0.001 compared to the Air group, as determined by the Mann–Whitney U test. (**F**) Heatmaps of Spearman’s rank correlation between the abundance of the changed gut microbiota and the levels of changed colon parameters in 3R4F-exposed mice (left) and Mb18W- and Mb30W-exposed mice (right).

The Chao1, Evenness, Simpson, Shannon, faith-pd, and observed otus indexes were used to reflect the α-diversity, i.e., the within-sample diversity, which estimates microbiome diversity within a single sample. None were significantly different among the groups (Figure 7A and Supplementary Figure S3). Indexes of β-diversity, i.e., the between-sample diversity, which estimates the microbiome similarity or dissimilarity between groups, were analysed. The phylogenetic-based distances for weighted and unweighted UniFrac did not show compositional difference of the microbiota among the four groups. By contrast, when the bacterial composition of the four groups was subjected to Bray–Curtis analysis, which does not account for phylogenetic relationships, there was a significant difference for the Mb18W-, Mb30W-, and 3R4F-exposed groups compared with the Air group (Figure 7B; *p* = 0.002, 0.001, and 0.017, respectively). Similar results were obtained with Jaccard analysis (Supplementary Figure S4). Analysis of ASV at the phylum level confirmed that the microbiome in all groups were dominated by *Bacteroidetes* and *Firmicutes* (Figure 7C). At the genus level, a drop in *Erysipelatoclostridium* and *Lachnospiraceae NK4A136* and a rise in *A2* relative abundance were observed in CS-exposed mice (Figure 7D). In mice chronically exposed to Mb18W inhalation, the relative abundance of *Parasutterella* was decreased (Figure 7E). The genus *Clostridia UCG-014* was more abundant in the microbiota of mice exposed to Mb18W and Mb30W.

In 3R4F-exposed mice, Spearman’ s correlation revealed significant associations between *Erysipelatoclostridium* relative abundance and *Ccnd1*, *Tgfb1*, *F11r* and *Cdh1* levels (*Ccnd1*: *r* = 0.6, *p* = 0.002; *Tgfb1*: *r* = 0.5, *p* = 0.01; *F11r*: *r* = 0.5, *p* = 0.01; *Cdh1*: *r* = 0.5, *p* = 0.01) (Figure 7F). *Lachnospiraceae NK4A136* was negatively correlated with the levels of *Cdkn2* (*r* = −0.5, *p* = 0.02) and *Ccnd2* (*r* = −0.5, *p* = 0.01), and positively correlated with the levels of *Foxp3* (*r* = 0.5, *p* = 0.01), *Ocln* (*r* = 0.5, *p* = 0.02), and *Cdh1* (*r* = 0.5, *p* = 0.01). A negative correlation between the *A2* and the colon mucosal surface was found (*r* = −0.5, *p* = 0.01). In e-cig-exposed mice, the relative abundance of *Parasutterella* was positively associated with the colon mucosal surface (*r* = 0.5, *p* = 0.01) and the mRNA levels of *Cdk2* (*r* = 0.6, *p* = 0.0002) and *Cdk4* (*r* = 0.5, *p* = 0.0009), and negatively correlated with *Egf* transcripts (*r* = −0.5, *p* = 0.001) and Ocln protein (*r* = −0.6, *p* = 0.03).

## 4. Discussion

The use of e-cigarettes is increasing among both adolescents and adults. E-cigarettes are popular alternative to conventional cigarettes, both among smokers and those who have never smoked. The health impact of CS exposure is well recognised, although the mechanisms remain insufficiently understood. Furthermore, the effects of e-cigarette use on the intestinal tract are only just beginning to be explored. In this study, we assessed the effects of chronic exposure to e-cig and CS on several major parameters of intestinal homeostasis in mice. Because region-specific modulations of intestinal response to smoke exposure has been described in the literature, we analysed the ileum and colon concomitantly [14,38].

In the ileum, a mild decrease in crypt depth and a small increase in villus length was observed, leading to a significant increase in villus/crypt ratio in mice exposed to high-power e-cig and to 3R4F CS. Fricker at al., who exposed female mice to the smoke of 12 3R4F cigarettes twice/day and 5 times/week for 12 weeks, observed a strong increase of villus length [13]. Relative to our study, the duration of exposure was shorter and experimental differences existed in terms of intensity of exposure, strain, and sex of the mice. The increase in the villus/crypt ratio that we observed was not associated with either increased apoptosis or cell proliferation. Biomarkers of several pathways of proliferation were disturbed, either up or down, and quite similarly between Mb30W and 3R4F exposure. Expression of the major protein of the cell cycle pathway, cyclin D1, was significantly higher in these two conditions. Overall, the ileum of mice exposed to Mb30W and 3R4F showed several impairments of proliferative signalling, which was not sufficient to induce a measurable cell overproliferation after 6 months’ exposure. Further studies are needed to decipher the long-term impacts in the ileum. By contrast, in the colon, CS had no effect on apoptosis or cell proliferation, whereas both Mb18W and Mb30W e-cig exposure induced evident limitation of cell proliferation, demonstrated by histological crypt loss, epithelial surface decrease and a reduced number of proliferative cells in crypts. According to our results, a decrease in colon cell proliferation was reported in mice exposed to e-cig liquid vapor [24]. In contrast, to our knowledge, CS was reported as promoting or inhibiting colon cancer-cell proliferation [21,39,40,41], and its effect on normal colonic mucosa has never been studied before. As in the ileum, several markers of proliferation pathways were disturbed by e-cig and CS exposure, but in the colon all were decreased. Further exploration of the cyclin D1 pathway showed up and down transcriptional dysregulations, which lead to a significant decrease in cyclin D1 protein levels. The cell cycle pathway is dysregulated in the ileum and colon by both e-cig and CS chronic exposure, and it may be one contributing factor of e-cig-induced cell proliferation decrease in the colon. Other contributing mechanisms remain to be identified.

Since accumulating evidence shows that CS modulates colon inflammation, we examined the effects of chronic e-cig and CS exposure on ileum and colon inflammatory markers. The response of the ileum to e-cig exposure was generally weak. However, e-cig induced upregulation of *Cxcl11*, *Cxcl16*, and *Cx3cr1*, which may contribute to recruitment of neutrophils, T cells and macrophages, respectively. Downregulation of Th2, Th17, and Treg cell transcription factors, as well as *Il1a*, *Il1b*, *Il6*, *Il18*, *Tnf*, *Ifng*, *Tgfb*, *Gzmb*, *Ccl2* and *Cxcl11* in the colon by e-cig exposure, was observed. These findings are in accordance with previous observations showing that inhalation exposure to flavoured e-cig JUUL Mango aerosols for three months resulted in downregulation of *Tnf*, *Il6*, and *Il1b* in the colon [25]. These data support an immunomodulatory effect of chronic e-cig exposure on the colon. However, upregulation of *Il4*, *Il9*, *Il12a*, *Il13*, *Il25*, *Ifnb1*, *Lta*, *Ccl22*, *Cxcl1*, and *Cxcr1* observed in Mb30W-exposed mice showed that the immune response is both activated and inhibited.

The response of the ileum to chronic exposure to CS was dominated by the drastic upregulation of *Cyp1a1* expression. It was associated with the upregulation of numerous inflammatory markers, including *Il7*, *Il15*, *Il33*, *Csf2*, *Pdgfa*, *Ccl22*, *Cxcl11*, and *Cxcl16*, compensated only by the drop in *Il1b* expression. These data support a weak immunostimulatory effect of chronic CS in the ileum. In the colon, our results showed significant modulation of numerous markers, which can inhibit as much as activate the immune response. They are in accordance with the contrasting effects of CS exposure described to date. Conventional smoking has been shown to have a protective effect on human UC [3,5] and in murine dextran sulfate sodium (DSS)-induced colitis [8,42,43]. Under inflammatory conditions in the colon, modulation of TLR and IFN pathways in iNKT lymphocyte, CD4^+^ T cell, and B cell subpopulations have been shown to contribute to smoke-induced colitis improvement. By contrast, a 4-day pre-exposure to CS in rats significantly potentiated colonic damage in a 2,4,6-trinitrobenzenesulfonic acid (TNBS) experimental model of colitis [44]. Consistently, a 4-week smoke exposure predisposes mice to more severe TNBS-induced colitis [13]. These contrasting effects on the colon immune response remain poorly understood.

By comparing the immune profiles between the two tissues and the two types of exposure, the colon was more sensitive than the ileum. In the colon, many targets were modulated at a similar level by CS and e-cig, and several additional targets were disrupted only by e-cig, suggesting that the disturbance of the colon immune response by e-cig was as severe as those by CS.

Both e-cig and CS reduced expression of tight junction proteins in the ileum and colon, which was confirmed by decreased ZO-1 and occludin expression in the ileum and colon, respectively. In accordance with our results, several studies have demonstrated a depletion of tight junctions following CS exposure, although the data are not always consistent. Exposure to CS for 10 weeks in mice depleted claudin-1, occludin, and ZO-1 in the small intestine, but did not affect these proteins in the colon [14]. Rat exposure for 14 weeks to CS did not modify the expression of occludin and Tjp-1 in the ileum, but down-regulated claudin-1 [45]. Also, chronic exposure to e-cig reduced occludin, ZO-1, and claudin-2 gene transcription in the distal mouse colon, and this diminution of tight junctions was confirmed in human colonoids [23]. Therefore, accumulating evidence argues in favour of a disruptive effect of conventional and electronic cigarette exposure on gut barrier function, both in the small intestine and the colon.

Since tight junctions are essential to maintain the cohesion and impermeability of the intestinal epithelium, a reduction in tight junctions by smoke exposure could disturb the homeostasis of gut microbiota and thus favour dysbiosis. Gut microbiota has a key role in gut-associated lymphoid tissue maturation and development, and there is communication with local immune cells to shape specific responses by balancing the tolerance and effector immune functions with various antigens [46]. A weakened gut barrier also promotes the translocation of pathogens through the intestinal mucosa and can increase susceptibility to infection. Moreover, beyond the gut, a unique coordination between the intestinal microbiota and liver, lung, and brain function exists. Indeed, dysbiosis has been implicated in various chronic metabolic and inflammatory diseases, systemic autoimmune disorders, and neurodevelopmental disorders [47,48,49]. Additionally, abnormalities of microbiota have been associated with development and progression of airway inflammatory diseases including chronic obstructive pulmonary disease, a major health problem that can result from smoking [50].

Because of the relationships between the gut immune response, permeability, and microbiota, we assessed the effects of e-cig and CS exposure on gut microbiota. A 6-month CS exposure did not lead to changes in α-diversity and bacterial composition at the phylum level. It did lead to a decreased abundance of two bacterial genera, *Erysipelatoclostridium* and *Lachnospiraceae NK4A136*, and to an increase in bacterial genus *A2*. Bacteria of the *Erysipelatoclostridium* genus are opportunistic pathogens and considered harmful bacteria. Their abundance is increased by various high-fructose [51,52] or high-fat [53,54,55] diets in mice, but is also decreased in constipated mice [56]. The *Lachnospiraceae NK4A136* group is considered a probiotic strain. Its relative abundance is reduced in DSS-induced colitis [57,58,59], in acetic acid-induced colitis [60], and in lipopolysaccharide (LPS)-induced intestinal mucosal injury [61], as well as in obese [62,63,64,65] and peanut-allergic mice [66]. *A2* strain also belongs to the *Lachnospiraceae* family, but is less known. Its enhanced abundance is correlated with improvement in repetitive behaviour and increased T reg cells in mesenteric lymph nodes in a mouse model of an autism spectrum disorder [67].

The extended heterogeneity of experimental protocols makes it difficult to compare these results with those of the literature. Several instances of smoke-induced dysbiosis were observed in pathological conditions, namely in mice with chemically induced colitis [43] and colorectal cancer [21], or in mice fed with a high-fat diet [68]. These pathological conditions drastically alter gut microbiota, which makes the comparison of the dysbiosis features irrelevant to steady-state conditions. As in this study, several studies of smoke-induced gut dysbiosis were conducted under non-pathological conditions. The indexes that we used concordantly showed that α-diversity was not modified by exposure to CS. Consistently, Shannon, ACE, and Chao1 indexes were unchanged after 0.5-, 1.5-, and 3-months CS exposure, respectively [69,70,71]. These latter α-diversity indexes were also found unmodified in colon microbiota after a three- [72] or 6-month exposure in addition to richness, Evenness, and Faith’s phylogenetic diversity [73].

Regarding the composition of bacterial microbiota at the phylum and genus levels, the changes induced by CS described in the literature are very heterogeneous. Overall, the type and number of genera disturbed by CS exposure shows that CS-induced dysbiosis is highly dependent on the experimental protocol. Taken together, studies performed in animals show that exposure to CS causes disturbances in the intestinal microbiota, which depend on numerous experimental parameters such as the murine strain, the type of microbiota analysed (caecal content, ileal or colon luminal content), the type of cigarette used, its concentration, exposure mode, and duration.

In humans, the effects of smoking on gut microbiota have been systematically evaluated [18]. A decrease in bacterial species diversity and reduced variability indexes in smokers’ faecal samples were found in most studies. Concerning the relative abundance at the phylum and genus levels, the results presented important heterogeneity both in smokers and non-smokers. However, *Proteobacteria* showed a progressive rise in *Desulfovibrio* with the number of pack-years of cigarette smoking and an increase in *Alphaproteobacteria* in current smokers versus never-smokers. *Prevotella* spp. appears to be significantly higher in smokers and former smokers. Finally, despite the numerous studies carried out, our knowledge of the health effects of dysbiosis induced by cigarette smoking and their mechanisms remain insufficiently understood and will still need to be deepened.

Regarding the effects of e-cig on gut microbiota, only one small human study has been conducted and concluded on the absence of differences in the α- and β-diversity or taxonomic relative abundances between e-cigarette users and controls [74]. No studies have been reported in animals. In our experimental conditions, chronic exposure to e-cig at the lower 18W power induced a severe decrease in *Parasutterella* relative abundance. *Parasutetterella*, a genus of *Betaproteobacteria*, is a core component of the human and mouse microbiota, which has been associated with different health outcomes such as IBD, obesity, diabetes, and fatty liver disease [75]. *Parasutterella* has a potential role in bile acid maintenance and cholesterol metabolism. Therefore, the drop in *Parasutterella* after low-power e-cig exposure could have health outcomes which deserve further investigation. *Parasutterella* abundance was positively correlated with colon mucosal surface, and colon *Cdk2* and *Cdk4* mRNA expression, suggesting its mediating role in e-cig-induced colon proliferation disturbances. Furthermore, e-cig chronic exposure at both 18W and 30W power enhanced the relative abundance of *Clostridium UCG-014.* Decreased abundance of *Clostridia UCG-014* has been reported in numerous pathological murine models, including DSS-induced colitis [76,77,78,79], in LPS-induced intestinal mucosal injury [61], temozolomide-induced intestinal mucositis [59], cyclophosphamide-induced immunosuppression [80] and high-fat fed mice [65,81]. By contrast, *Clostridia UCG-014* was found to be related to the development of rheumatoid arthritis [82], and increased in colitis-associated colorectal cancer [83].

Despite the accumulating literature and the new knowledge brought by this study, how CS and e-cig compromise gut health is incompletely understood. Nevertheless, it is known that CS and e-cig have both a direct and indirect impact on the ileum and colon. CS and e-cig are composed of gas and particulate phases and contain hundreds of substances [84]. Substances in the gas phase, such as carbon monoxide and light aldehydes, can pass through the airway epithelial barrier and enter the systemic circulation via the pulmonary circulation. On the other hand, compounds such as nicotine, polycyclic aromatic compounds, nitrosamines and heavy metals are predominantly found in the particulate phase, being absorbed by mucous membranes, skin, alveoli and the gastrointestinal system [85]. It is noteworthy that nicotine was shown to accumulate in the saliva and gastric juice in humans using nicotine skin patches, and in the ileal mucosa of smokers, and therefore could exert its well-known protective intestinal effects topically [84,86,87]. Moreover, exposure of murine- and huma- enteroid-derived monolayers to nicotine-free e-cigarette aerosols caused barrier disruption, suggesting that the barrier-disruptive injury could be a direct response of the intestinal epithelium to e-cig-emitted chemical compounds [23]. In addition, other identified mechanisms rather argue in favour of an indirect impact of CS and e-cig in the intestinal tissues. For instance, Kim et al. reported that the local lung Th17 response induced by CS led to increased intestinal pathology through enhanced circulating neutrophils [7]. Frickler et al. demonstrated that CS induced impaired gas exchange and pathology in the lung, which subsequently led to ischemic damage in the colon and ileum [13]. Overall, e-cig and CS are composed of multiple chemical compounds that may exert direct or indirect effects in the colon and ileum. Each of these compounds may cause similar or dissimilar effects. For example, nicotine has been shown to exert anti-inflammatory effects in the colon, as well as pro-inflammatory effects in the jejunum [88,89,90]. As previously described, the combination of direct and indirect effects of the multiple e-cig and CS compounds also results in contrasting effects in the colon and ileum. Accordingly, we observed opposing effects on cyclin D1 between the colon and ileum. The functional consequences of the tissue-specific adverse effects caused by e-cig exposure deserve further investigation, especially since they are demonstrated in the current study to be of similar magnitude to those of CS.

## 5. Conclusions

In summary, our results show that the effects of chronic e-cig and CS exposure are different between the ileum and the colon. E-cig and CS have common and specific effects in intestinal tissues. In the ileum, both e-cig and CS disturbed epithelium turnover and increased cyclin D1 expression. E-cig decreased cell proliferation of colon mucosa, whereas CS had no effect. E-cig and CS reduced tight-junction expression in the ileum and colon, and induced mild microbiota structural modifications. The mucosal immune response was dysregulated by e-cig and CS, at a higher extent in the colon than in the ileum. Overall, the impact on the ileum and colon appears as severe for e-cig as that which was observed for CS exposure. Therefore, e-cig exposure has the potential to cause gut health effects and contribute to intestinal and extra-intestinal diseases.

## Data Availability

The raw data supporting the conclusions of this article will be made available by the authors on request.

## Supplementary Materials

The following supporting information can be downloaded at: https://www.mdpi.com/article/10.3390/jox14030053/s1, Figure S1: Plasma cotinine levels in mice exposed for 6 months to Mb18W, Mb30W vapors and 3R4F CS; Figure S2: Effects of chronic e-cigarette and cigarette exposure on ileum (A) and colon (B) myeloperoxidase activity level; Figure S3: Effects of chronic e-cigarette and cigarette exposure on gut microbiota. α-diversity indexes; Figure S4: Effects of chronic e-cigarette and cigarette exposure on gut microbiota. Jaccard index; File Sl: Supplementary Methods. References [91,92,93,94] are cited in the Supplementary Materials.
