# Supplementary material for: Chronic Exposure to Both Electronic and Conventional Cigarettes Alters Ileum and Colon Turnover, Immune Function, and Barrier Integrity in Mice
Source: J Xenobiot. 2024 Jul 22;14(3):950–69. doi: 10.3390/jox14030053 (PMC11270428; doi:10.3390/jox14030053)
Supplement: Supplementary file 1 [file jox-14-00053-s001.zip › JoX-3059509 suppl data.pdf]

Supplementary Figures

Chronic Exposure to Both Electronic and Conventional Cigarettes Alters Ileum and Colon Turnover, Immune Function, and Barrier Integrity in Mice.

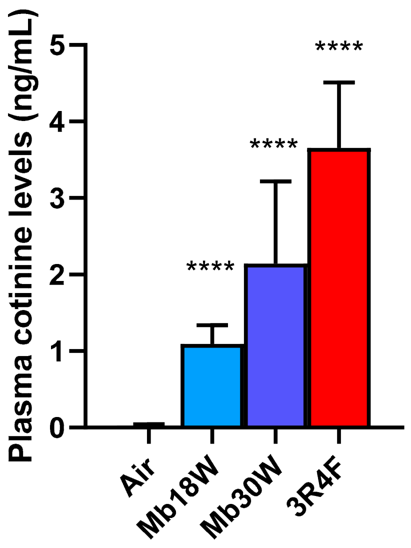

Figure S1: Plasma cotinine levels in mice exposed for 6 months to Mb18W, Mb30W vapors and 3R4F CS (n=14/group). ****p < 0.001 compared to the control group (Air) as determined by the Mann-Whitney U test.

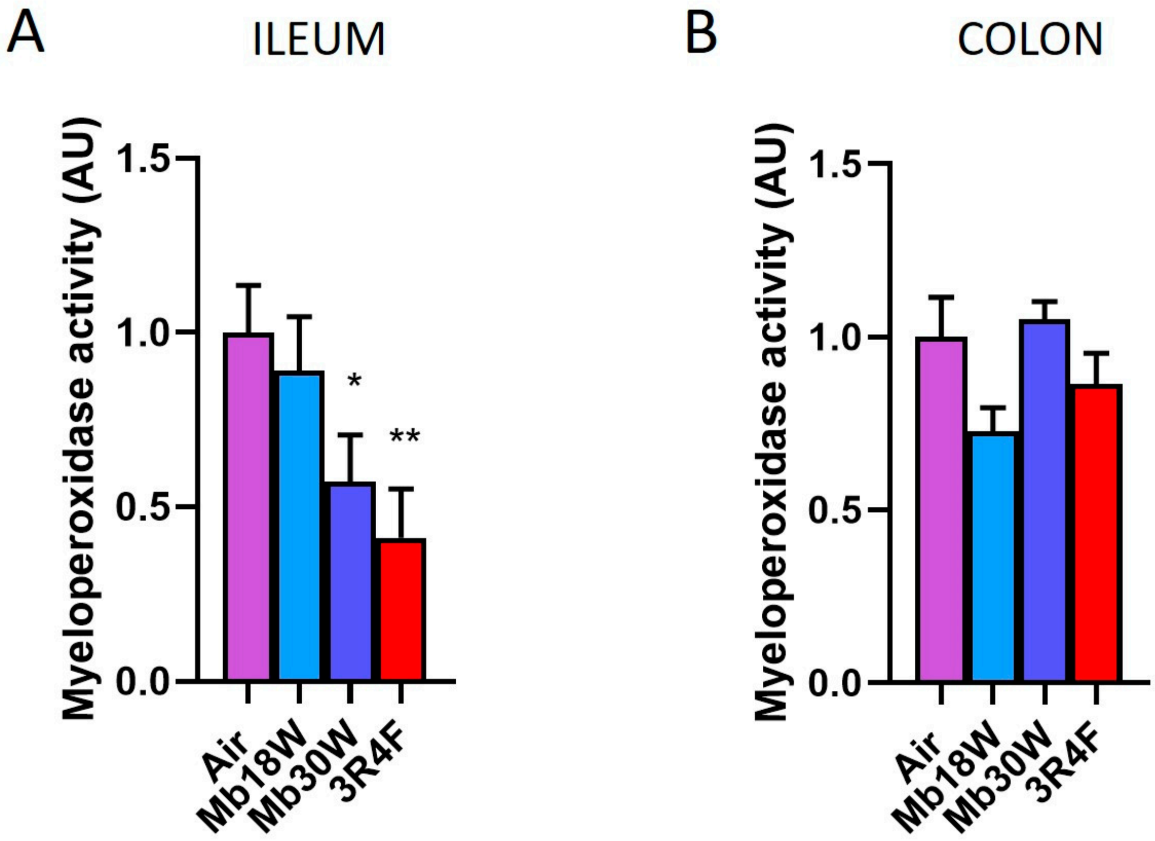

Figure S2: Effects of chronic e-cigarette and cigarette exposure on ileum (A) and colon (B) myeloperoxidase activity level. n=14 per group. *p<0.05, **p<0.01.

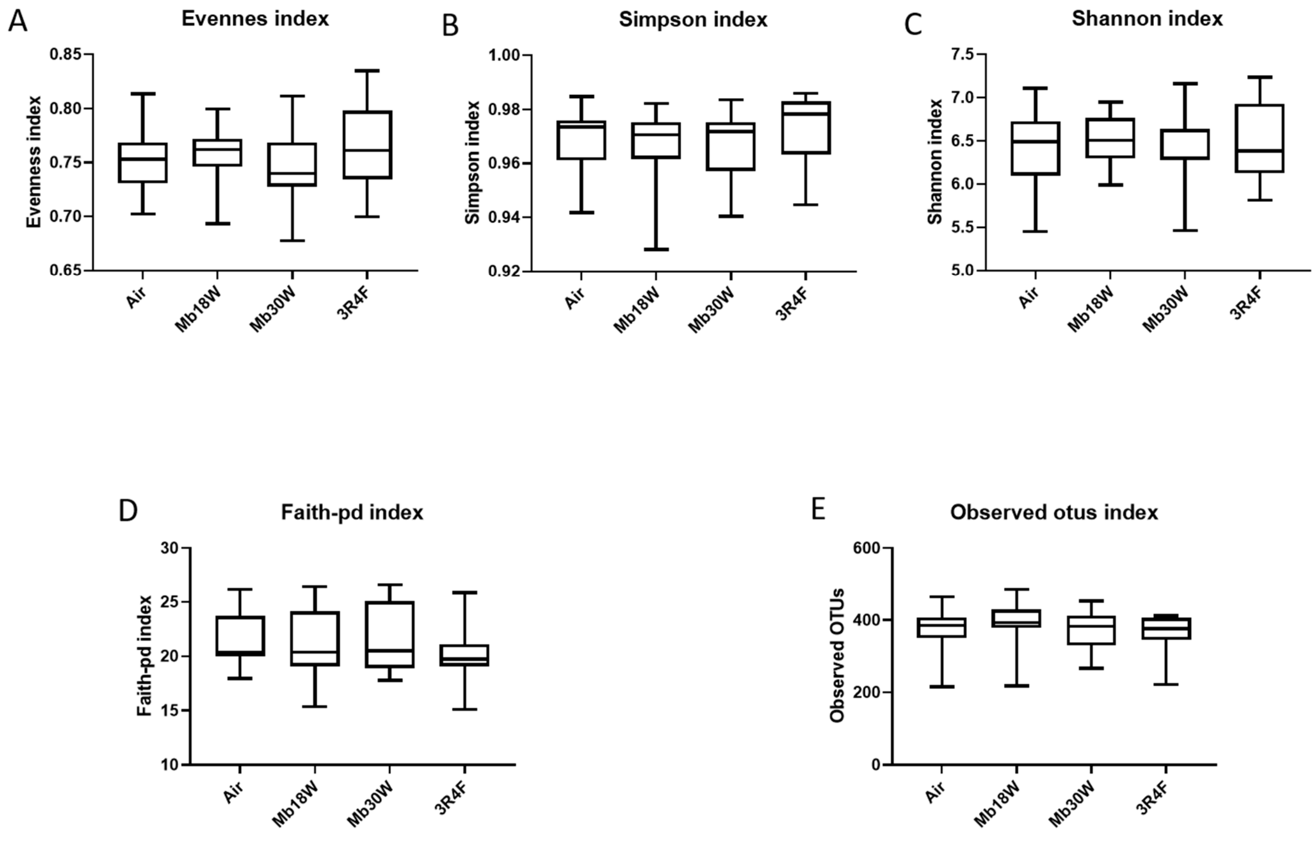

Figure S3: Effects of chronic e-cigarette and cigarette exposure on gut microbiota. α-diversity indexes. n=14 per group.

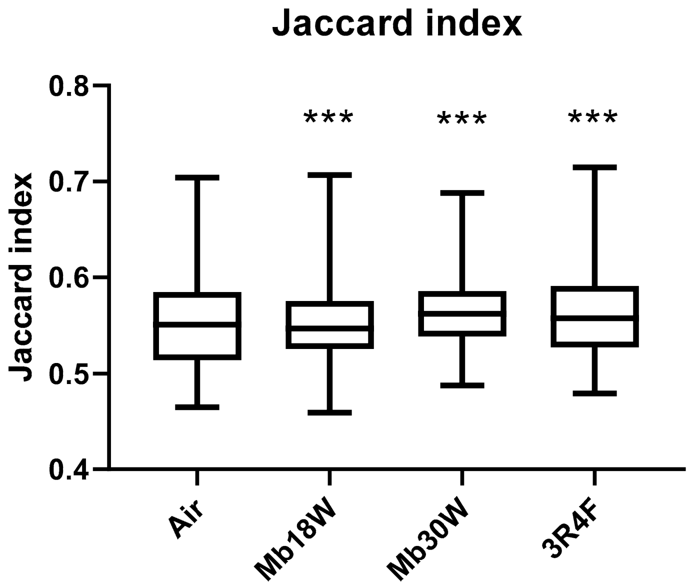

Figure S4: Effects of chronic e-cigarette and cigarette exposure on gut microbiota. Jaccard index. n=14 per group. ***p < 0.005 compared to the control group (Air) as determined by the Mann-Whitney U test.
