# Supplementary material for: Chronic Exposure to Both Electronic and Conventional Cigarettes Alters Ileum and Colon Turnover, Immune Function, and Barrier Integrity in Mice
Source: J Xenobiot. 2024 Jul 22;14(3):950–69. doi: 10.3390/jox14030053 (PMC11270428; doi:10.3390/jox14030053)
Supplement: Supplementary file 1 [file jox-14-00053-s001.zip › JoX-3059509- suppl file S1.pdf]

Supplementary methods

Chronic Exposure to Both Electronic and Conventional Cigarettes Alters Ileum and Colon Turnover, Immune Function, and Barrier Integrity in Mice.

Histological analysis

At necropsy, ileum and colon tissue were fixed in 4% formaldehyde overnight, processed, and embedded in paraffin wax by an automatic sample preparation system (LOGOS One, Milestone, Valbrembo, Italy). Serial histological sections of 4 µm thickness were cut, deparaffinised, rehydrated, and stained with May Grünwald Giemsa (MGG; Carlo Erba, Val de Reuil, France). Images were acquired with a DM5500 B microscope (Leica Microsystems, Wetzlar, Germany). Histomorphometric analyses were performed using Image J software (NIH). Epithelial area in the colon, villus height, and crypt depth in the proximal and distal small intestine were measured according to the scheme presented below. At least 100 well-oriented mucosa, villi, and crypts were measured in at least 5 individual mice from each group.

Scheme of histomorphometric measures

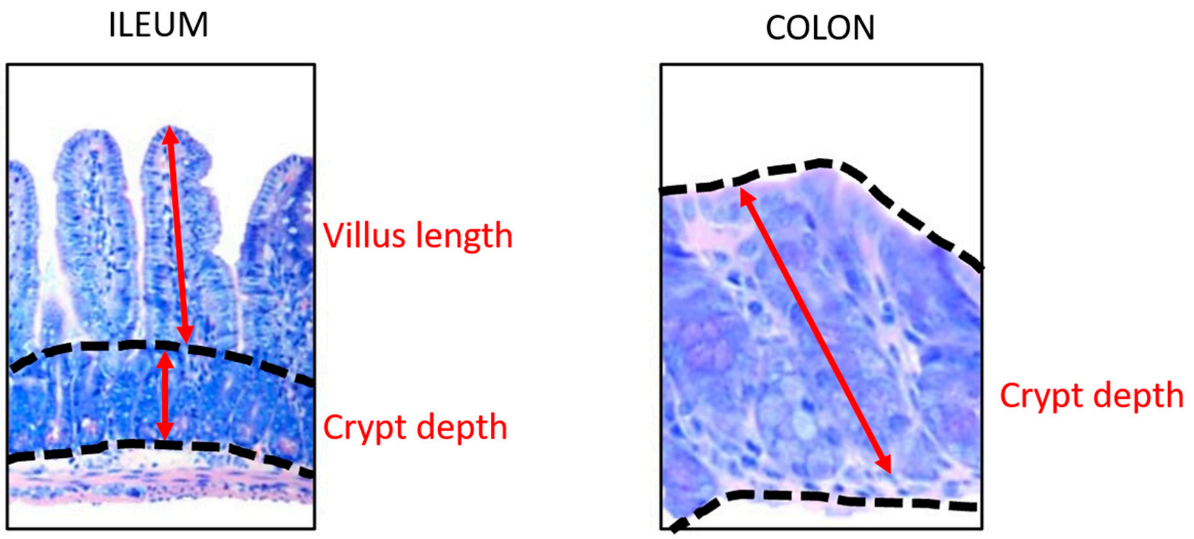

Quantitative RT-PCR

Small intestinal and colonic tissue samples were homogenised with ceramic beads using Precellys Lysing Equipment (Bertin Technologies, Montigny le Bretonneux, France). Total RNA was extracted with the NucleoSpin RNA kit (Macherey-Nagel, Hoerdt, France). Transcript levels of genes were quantified with the StepOne™ Real-Time PCR system using a SYBR Green PCR master mix (Thermo Fisher Scientific, Illkirch, France). The primer sequences were designed using the National Library of Medicine Primer Designing Tool and are presented in Table 1 below. Melting curve analyses were performed for each sample and gene to confirm the specificity of the amplification. Three reference genes were tested for this study: b-Actin, Gapdh, and PolR2a. Polr2A was found to be the most stable and showed no variation between treatments, so was chosen as reference gene. Additional targets were quantified using the TaqMan Fast advanced Master Mix and Taqman assays (Thermo Fisher Scientific, Illkirch, France) and are presented in Table 2. Quantification of target gene expression was based on the comparative cycle threshold (Ct) value. The fold changes in the target genes were analysed by the $2^{-\Delta\Delta Ct}$ method.

Bacterial DNA extraction and Illumina MiSeq sequencing

Genomic DNA was extracted from colon luminal content using the DNA Stool kit (Macherey Nagel, Hoerdt, France). The quantity and purity of DNA (expressed as the ratio of absorbance at 260 and 280 nm) were assessed using a NanoDrop® spectrophotometer (Ozyme, Saint-Cyr-l'école, France). The sequencing library was generated by amplifying the V3-V4 regions of the bacterial 16S-rRNA gene using 16S rRNA amplicon generation for MiSeq with the primers Bact-0341 (CCTACGGGNGGCWGCAG) and Bact-0785 (GACTACHVGGGTATCTAATCC). Individual samples were barcoded, pooled to construct

the sequencing library, and sequenced using an Illumina MiSeq system (Illumina, Evry, France) to generate paired-end 2x300 bp reads.

Table 1.

Gene	Primer sequences
mTbx21-F	CCAAAGGATTCCGGGAGAA
mTbx21-R	CTCGTATCAACAGATGCGTACATG
mTnf-F	CCA-CCA-CGC-TCT-TCT-GTC-TA
mTnf-R	GAG-GCC-ATT-TGG-GAA-CTT-CT
mIfng-F	ACTGGCAAAAGGATGGTGAC
mIfng-R	GCTGATGGCCTGATTGTCTT
mGata3-F	GCAGAACCGGCCCCTTAT
mGata3-R	ACAGTTCGCGCAGGATGTC
mIl4-F	CATGCACGGAGATGGATGTG
mIl4-R	TGGTGTTCTTCGTTGCTGTGAG
mIl13-F	CCA-ATT-GCA-ATG-CCA-TCT-ACA
mIl13-R	GGG-CCT-TGC-GGT-TAC-AGA
mIl25-F	GTACCAGGCTGTTGCATTCTTG
mIl25-R	CTGACGGTGTGGGTTCCC
mIl9-F	TGT-GTG-CTG-CAT-GAC-CAA-CAA
mIl9-R	GGC-AAT-GCT-GCT-GAT-TCT-CCT
mRorc-F	CTTCCTCAGCGCCCTGTGT
mRorc-R	TGAGAACCAGGGCCGTGTAG
mFoxp3-F	CAACATGGACTACTTCAAGTACCACAATA
mFoxp3-R	GATGGCCCATCGGATAAGG
mIl10-F	CCC-TTT-GCT-ATG-GTG-TCC-TT
mIl10-R	TGG-TTT-CTC-TTC-CCA-AGA-CC
mTgfb1-F	CCC GAA GCG GAC TAC TAT GCT
mTgfb1-R	GTT TTC TCA TAG ATG GCG TTG TTG
mIl12a-F	CGAGACTCTGCGCCAGAAAC
mIl12a-R	GCAGGATGCAGAGCTTCATTT
mIl1a-F	GCTTGAGTCGGCAAAGAAATC
mIl1a-R	GAGAGAGATGGTCAATGGCAGA
mIl1b-F	AGCTCTCCACCTCAATGGAC
mIl1b-R	AGGCCACAGGTATTTTGTCG
mIl18-F	CACTTCTCCCCTGTGGTGTG
mIl18-R	GCTGTGCCAGTCTGCAGTT
mIL6-F	TACACATGTTCTCTGGGAAATCGT
mIL6-R	AAGTGCATCATCGTTGTTCATACA
mRetnla-F	CCCTGCTGGGATGACTGCTA
mRetnla-R	CTCCACTCTGGATCTCCCAAGA
mVegfa-F	AAC-GAT-GAA-GCC-CTG-GAG-TG
mVegfa-R	GCT-GGC-TTT-GGT-GAG-GTT-TG

mGzmb-F	CAGCAAGTCATCCCTATGGTAAA
mGzmb-R	TCTAGTCCTCTTGGCCTTACTC
mCcl2-MCP1 F	AGGTCCCTGTCATGCTTCTG
mCcl2-MCP1 R	TCTGGACCCATTCCTTCTTG
mCcl3-MIP1a F	AGATTCCACGCCAATTCATC
mCcl3-MIP1a R	CTCAAGCCCCTGCTCTACAC
mCcl5-Rantes-F	GTGCTCCAATCTTGCAGTCGT
mCcl5-Rantes-R	ACACACTTGGCGGTTCCTTC
mCcl20-F	CGA-CTG-TTG-CCT-CTC-GTA-CA
mCcl20-R	AGG-AGG-TTC-ACA-GCC-CTT-TT
mCxcl1-F	GGCGCCTATCGCCAATG
mCxcl1-R	CTGGATGTTCTTGAGGTGAATCC
mCyp1a1-F	CCA-CCT-GCT-GAG-GCT-AAA-CAG
mCyp1a1-R	TGC-CCC-CCA-CAT-GCA
mCyp2a4-F	TCG-AGG-AGC-GCA-TCC-AA
mCyp2a4-R	AAT-GAA-AGC-ACC-GTT-CGT-CTT-C
mPolR2a-F	CCCACAACCAGCTATCCTCAA
mPolr2a-R	GGTGCTGTGGGTACGGATACA

Table 2.

**Gene**	**Taqman assay ID**
CCL4/MIP-1 beta	Mm00443111_m1
CCL11/Eotaxin	Mm00441238_m1
CCL19/MIP-3 beta	Mm00839966_g1
CD40 Ligand/TNFSF5	Mm00441911_m1
CXCL2/GRO beta/MIP-2/CINC-3	Mm00436450_m1
CXCL10/IP-10/CRG-2	Mm00445235_m1
EGF	Mm00438696_m1
FGF basic/FGF2/bFGF	Mm01285715_m1
Flt-3 Ligand/FLT3L	Mm00442801_m1
G-CSF	Mm00438334_m1
GM-CSF	Mm01290062_m1
IFN-alpha 2/IFNA2	Mm00833961_s1
IFN-beta	Mm00439552_s1
HMOX1	Mm00516005_m1
Cyp4f18	Mm07298284_m1
Cyp2a5	Mm00487248_g1
Txnrd1	Mm00443675_m1
Mt2	Mm00809556_s1
Cyp1a1	Mm00487218_m1
Aldh3a1	Mm00839312_m1
Ahrr	Mm00477443_m1
Nqo1	Mm01253561_m1

Cxcr1	Mm00731329_s1
Gpx2	Mm01286848_gH
Gsto1	Mm00599866_m1
IL-1ra/IL-1F3	Mm00434237_m1
IL-2	Mm00434256_m1
IL-3	Mm00439631_m1
IL-7	Mm01295803_m1
IL-15	Mm00434210_m1
IL-17/IL-17A	Mm00439618_m1
IL-33	Mm00505403_m1
Lymphotoxin-alpha/TNF-beta	Mm00440228_gH
PD-L1/B7-H1	Mm03048248_m1
TGF-alpha	Mm00446232_m1
TRAIL/TNFSF10	Mm01283606_m1
IL-22	Mm01226722_g1
CXCL3	Mm01701838_m1
CXCL5	Mm00436451_g1
CXCL11	Mm00444662_m1
CXCL16	Mm00469712_m1
CCL22	Mm00436439_m1
PDGF-A	Mm01205760_m1
PDGF-B	Mm00440677_m1
CCL28	Mm00445039_m1
CX3CL1	Mm00436454_m1
RPLP0	Mm00725448_s1
PPIA	Mm02342430_g1

Analysis of sequencing data

Bioinformatic analyses were performed using the QIIME2 pipeline (ver. 2020.2) [91]. The Divisive Amplicon Denoising Algorithm 2 (DADA-2) plug-in in QIIME2 was used to filter, dereplicate, identify chimeric sequences, and merge reads to obtain the set of amplicon sequence variants (ASVs) for each sample [92]. Then the representative sequences were picked for each ASV. The classify-sklearn plug-in in QIIME2, with the SILVA database (ver. 132), was applied to assign a taxonomic annotation to each representative ASV sequence. In the next step, ASVs identified as eukaryotic contamination (3 ASVs; 12 reads) and external contamination, identified with the decontam package (3 ASVs; 3119 reads), were filtered out

[93]. Diversity metrics (α and β) were obtained with the QIIME2 core-metrics-phylogenetic plug-in, with p-sampling depth parameter fixed to 13781 reads which corresponded to the total frequency that each sample should be rarefied to prior to computing diversity metrics. This sampling depth allowed retention of >61% of reads and the discarding of only one sample. Tests for differential relative abundance were performed with corncob at the order, family, and genus levels [94].Spearman's correlation were performed using Prism 8 software (GraphPad Prism software v8).

References

91. Bolyen, E.; Rideout, J.R.; Dillon, M.R.; Bokulich, N.A.; Abnet, C.C.; Al-Ghalith, G.A.; Alexander, H.; Alm, E.J.; Arumugam, M.; Asnicar, F.; et al. Reproducible, Interactive, Scalable and Extensible Microbiome Data Science Using QIIME 2. *Nat. Biotechnol.* **2019**, *37*, 852–857, doi:10.1038/s41587-019-0209-9.
92. Callahan, B.J.; McMurdie, P.J.; Rosen, M.J.; Han, A.W.; Johnson, A.J.A.; Holmes, S.P. DADA2: High-Resolution Sample Inference from Illumina Amplicon Data. *Nat. Methods* **2016**, *13*, 581–583, doi:10.1038/nmeth.3869.
93. Davis, N.M.; Proctor, D.M.; Holmes, S.P.; Relman, D.A.; Callahan, B.J. Simple Statistical Identification and Removal of Contaminant Sequences in Marker-Gene and Metagenomics Data. *Microbiome* **2018**, *6*, 226, doi:10.1186/s40168-018-0605-2.
94. Martin, B.D.; Witten, D.; Willis, A.D. Modeling Microbial Abundances and Dysbiosis with Beta-Binomal Regression. *Ann. Appl. Stat.* **2020**, *14*, 94–115, doi:10.1214/19-aoas1283.
